# MIST1, an Inductive Signal for Salivary Amylase in Mesenchymal Stem Cells

**DOI:** 10.3390/ijms20030767

**Published:** 2019-02-12

**Authors:** Mahmoud Mona, Rehae Miller, Hui Li, Yun-Jong Park, Raafi Zaman, Li-Jun Yang, Seunghee Cha

**Affiliations:** 1 Oral and Maxillofacial Diagnostic Sciences, University of Florida College of Dentistry, Gainesville, FL 32610, USA; mmona@dental.ufl.edu (M.M.); RMiller2@dental.ufl.edu (R.M.); raafiz@ufl.edu (R.Z.); 2 Oral Biology, University of Florida College of Dentistry, Gainesville, FL 32610, USA; 3 Pathology, University of Florida College of Medicine, Gainesville, FL 32610, USA; lih@ufl.edu (H.L.); yanglj@pathology.ufl.edu (L.-J.Y.); 4 Pharmacology, University of North Carolina College of Medicine, Chapel Hill, NC 27599, USA; yjpark7604@gmail.com

**Keywords:** mouse bone-marrow-mesenchymal stem cells, alpha-salivary amylase 1, MIST1, TCF3, Sjögren’s syndrome, salivary precursors

## Abstract

Sjögren’s syndrome (SjS) is an autoimmune disease that destroys the salivary glands and results in severe dry mouth. Mesenchymal stem cell (MSC) transplantation has been recently proposed as a promising therapy for restoring cells in multiple degenerative diseases. We have recently utilized advanced proteomics biochemical assays to identify the key molecules involved in the mesenchymal-epithelial transition (MET) of co-cultured mouse bone-marrow-derived MSCs mMSCs with primary salivary gland cells. Among the multiple transcription factors (TFs) that were differentially expressed, two major TFs were selected: muscle, intestine, and stomach expression-1 (MIST1) and transcription factor E2a (TCF3). These factors were assessed in the current study for their ability to drive the expression of acinar cell marker, alpha-salivary amylase 1 (AMY1), and ductal cell marker, cytokeratin19 (CK19), in vitro. Overexpression of MIST1-induced AMY1 expression while it had little effect on CK19 expression. In contrast, TCF3 induced neither of those cellular markers. Furthermore, we have identified that mMSCs express muscarinic-type 3 receptor (M3R) mainly in the cytoplasm and aquaporin 5 (AQP5) in the nucleus. While MIST1 did not alter M3R levels in mMSCs, a TCF3 overexpression downregulated M3R expressions in mMSCs. The mechanisms for such differential regulation of glandular markers by these TFs warrant further investigation.

## 1. Introduction

Autoimmune Sjögren’s syndrome (SjS) is the third most common autoimmune disease after rheumatoid arthritis (RA) and systemic lupus erythematosus (SLE) [1,2]. SjS and radiation therapy for head and neck cancer cause the destruction of the salivary gland acinar cells. The damage to the salivary glands, especially acinar cells, leads to reduced salivary flow resulting in severe dry mouth (xerostomia) [3,4]. Acinar cells are responsible for secreting water, electrolytes, mucus, antibacterial components, and enzymes, especially, the carbohydrate digestive enzyme, salivary amylase 1(AMY1) [5]. AMY1 helps break down carbohydrate, which yields simple sugars, such as dextrin and maltose, which are then further broken down into simpler sugars in the small intestine to be absorbed into the bloodstream [6]. Individuals with proper levels of AMY1 can rapidly digest starch and better maintain balanced blood sugar. In addition, xerostomia may result in severe dental caries and periodontitis along with difficulty in chewing and swallowing [3,7]. According to the Sjögren’s Syndrome Foundation, four million Americans have been diagnosed with SjS [8]. Current treatment for SjS or radiation-induced xerostomia is limited to the stimulation of remaining acinar cells by secretagogues [9], or palliative therapies such as artificial saliva [10], all of which do not appear to significantly improve salivary flow due to the permanent damage to the salivary acinar cells [11].

Lack of effective therapy for this devastating condition has led researchers to explore novel and promising cellular regeneration therapies [12]. Studies on the stem-cell regeneration of various organs, such as the liver, pancreas, muscles, neurons, and many other tissues, have given hope to many patients suffering from multiple degenerative diseases [13,14,15]. However, the hostile microenvironment of the damaged salivary gland is assumed to hinder potential resident stem cell differentiation in situ [16,17]. Thus, the selection of proper stem cells and the ability to genetically engineer those stem cells so that they promote their own differentiation in the unfavorable microenvironment have become crucial. Stem cells could originate from multiple sources: embryonic, stromal stem cells, mesenchymal stem cells (MSCs), or induced pluripotent stem cells [18]. The most commonly used are MSCs [19], which are multipotent cells [20]. They have the ability to differentiate into adipocytes [21], chondrocytes [22], osteoblasts [23], and myoblasts [24]. More importantly, MSCs have proved to be capable of differentiating into exocrine cells, such as those found in the mammary glands, the liver, and pancreas [25,26,27].

As previously shown by our laboratory, mouse bone marrow-derived MSCs (mMSCs) have the ability to transdifferentiate into salivary precursors in a co-culture system [28]. Without cell-to-cell contact, mMSCs transdifferentiated into salivary precursors with clustered morphology resembling primary salivary epithelial cells after seven days of co-culture [28]. Proteomic analyses by our group have identified transcription factors (TFs) that are differentially upregulated in transdifferentiating mMSC, namely, pancreas-specific transcription factor 1 α (PTF1α), muscle, intestine, and stomach expression-1 (MIST1), transcription factor E2a (TCF3), achaete-scute complex homolog 3 (ASCL3), ankyrin repeat domain-containing protein 56 (ANKRD56), and high mobility group protein 20B (HMG20B) [2]. Of those selected TFs, MIST1 and TCF3 are basic helix-loop-helix (bHLH) transcriptional regulators. They form a homodimer or heterodimer to bind to E-box in DNA and activate several regulatory genes. It was shown recently that MIST1 alone induces and maintains pancreatic acinar cell secretory phenotype while TCF3 plays a major role in cellular development and differentiation.

In this study, we discovered for the first time that MIST1 induces the AMY1 gene and protein expression whereas TCF3 overexpression exerts a suppressive effect on the mMSC-to-salivary precursor differentiation process. Furthermore, we also detected the protein expression of aquaporin-5 (AQP5) and muscarinic type 3 receptor (M3R), which are important salivary gland acinar cell markers for salivation [29], in mMSCs. AQP5 is a water channel protein that is abundantly expressed in the salivary glands [30]. It is specifically expressed in the basolateral membranes of acinar cells and plays a major role in fluid secretion [31,32]. It has also been shown to be involved in tear production and pulmonary secretions [32]. M3R is a G protein-coupled receptor expressed in salivary gland cells that responds to acetylcholine stimulation for saliva secretion [33,34]. Interestingly, they were irresponsive to secretory stimulation; this was potentially due to their intranuclear and intracytoplasmic localizations, respectively. In our present study, we examine the functional roles of MIST1 and TCF3 in regulating the expression of AMY1 and ductal cytokeratin 19 (CK19) in mMSCs during the mesenchymal-epithelial transition (MET) process and explore the characteristics and properties of mMSCs for the expression of important acinar marker proteins for secretion, such as M3R and AQP5.

## 2. Results

### 2.1. Recombinant MIST1 or TCF3 Expression Was Confirmed in mMSCs

**Figure 1:**
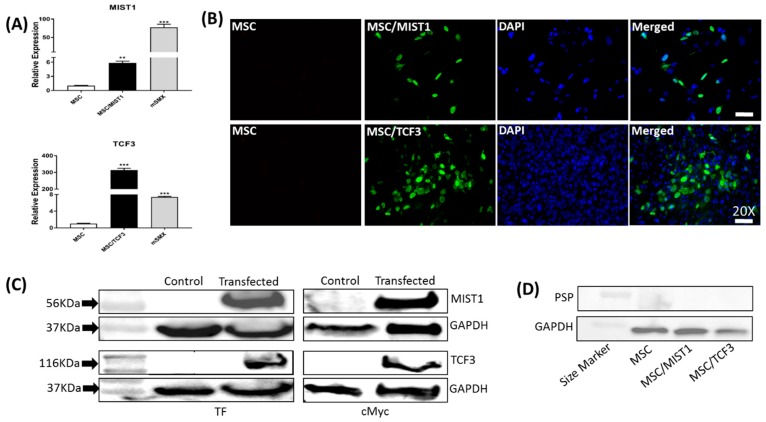
Confirmation of recombinant MIST1 and TCF3 expression by ICC, WB and qRT-PCR. (**A**) qRT-PCR was used to confirm the gene expression of recombinant MIST1 and TCF3 in transfected mouse bone-marrow-derived mesenchymal stem cell (mMSCs). The expression was absent in untransfected mMSCs as expected. Mouse submandibular gland lysate (mSMX) was used as a positive control. (**B**) ICC was performed for TCF3 and MIST 1 on transfected mMSCs to confirm protein expression. We used 4′,6-diamidino-2-phenylindole (DAPI) to stain nuclei. Images demonstrate a 28–34% transfection efficiency. Merged images confirm nuclear localization of the transcription factors (TFs). (Magnification, × 20; scale bar, 20 μm). (**C**) Transfection was confirmed by the detection of cMyc tag and TFs (MIST1 or TCF3) in the protein lysates collected at 24-h post-transfection. Bands correspond to the sizes of 56kDa for MIST1 and 116kDa for TCF3 in transfectants whereas endogenous expression of MIST1 or TCF3 was absent in untransfected mMSCs. Each well was loaded with 30 µg of protein. GAPDH protein was used as a loading control (lower panels). (**D**) PSP expression was not detected in the MIST1 or TCF3 transfected mMSCs. Asterisks ** and *** indicate statistical significance levels of *p* < 0.01 and *p* < 0.001, respectively.

To analyze the effects of MIST1 and TCF3 overexpression on mMSC transdifferentiation into salivary precursors, pcDNA3.1cmycBioID plasmid expressing MIST1 or TCF3 was introduced into mMSCs by transient transfection utilizing the Lipofectamine-Stem system (Thermo Fisher Scientific, Waltham, MA, USA). Gene expression was confirmed first by qRT-PCR (Figure 1A). MIST1 transfection resulted in MIST1 overexpression with a six-fold increase while TCF3 transfection increased its expression by 300-fold compared to the control. The mRNA levels were compared to the level of expression in an 8-week old mouse submandibular gland lysate where the TFs are known to be expressed. At 24-h post-transfection, we performed Immunocytochemistry (ICC) to detect the recombinant MIST1 and TCF3 protein expression in mMSCs. The ICC was conducted more than three times and we detected the proteins of interest in the nucleus as expected (Figure 1B). Transcription efficiency ranged from 10% to 34%, with a maximum achieved of 34% for MIST1 and 28% for TCF3, as shown in Figure 1B, with strong protein expression in the transfection-positive cells. Western blotting (WB) was also performed to confirm MIST1 and TCF3 protein expression levels in the respective transfected mMSCs. As shown in Figure 1C, we detected the recombinant proteins at band sizes of 56 kDa for MIST1 and 116 kDa for TCF3, which includes protein tags. GAPDH on the second panel confirms an equal amount of loaded protein (Figure 1C). We initially examined the transfected cells to examine if MIST1 or TCF3 can lead to the expression of an acinar cell marker such as parotid secretory protein (PSP). As shown in Figure 1D, PSP was not induced by those factors.

### 2.2. MIST1 Promotes AMY1 in mMSCs Whereas TCF3 Does not Induce its Expression

**Figure 2:**
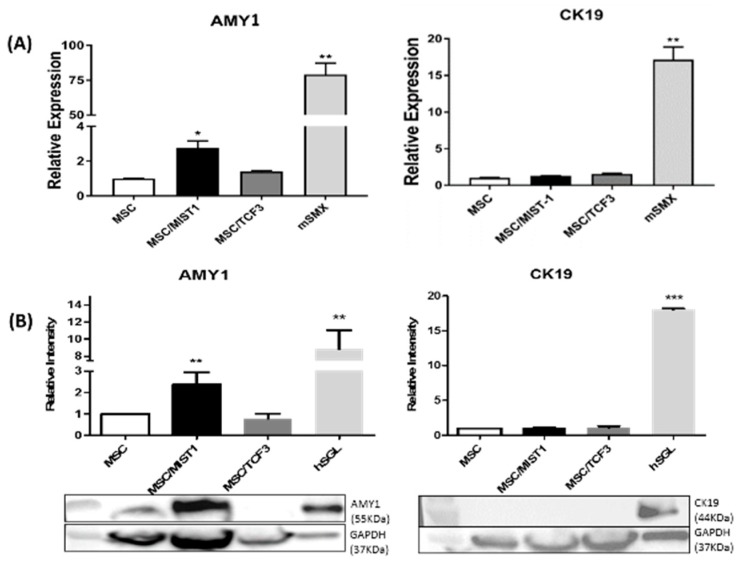
Protein and mRNA expression levels of AMY1 and CK19 in mMSCs in response to MIST1 and TCF3 overexpression. (**A**) qRT-PCR was performed to compare AMY1 and CK19 mRNA expression levels by purifying total RNA from mMSCs at 24 h post-transfection. Relative expression was calculated by the 2^−∆∆Ct^ method. The base level of gene expression in untransfected mMSCs was considered; MIST1 transfection has induced a 1.5-fold increase of AMY1 gene expression above the basal level. TCF3 transfection didn’t increase AMY1 or CK19 gene expression. Values were normalized to the amount of 18S mRNA. (**B**) MIST1 transfection of mMSCs have led to a 2.5-fold increase in AMY1 expression compared to the level of expression in untransfected mMSCs. Untransfected mMSCs were considered; TCF3 transfection didn’t influence AMY1 protein expression (55 kDa). CK19 protein (44kDa) expression was not altered by MIST1 or TCF3 overexpression in mMSCs. Human salivary gland lysate (hSGL) was used as a positive control. The intensity of each band was normalized for the intensity of GAPDH. For (**A**) and (**B**), experiments were repeated three times. Asterisks *, ** and *** indicate *p* < 0.05, *p* < 0.01 and *p* < 0.001, respectively. Error bars indicate means ± SEM.

At 24-h post-transfection with MIST1 or TCF3, we measured the mRNA levels of another acinar cell marker, AMY1, and a ductal cell marker, CK19, utilizing qRT-PCR. Levels of MIST1 and TCF3 mRNA were quantified utilizing primers specific for each gene. MIST1 transfected cells induced the expression of AMY1 mRNA by 150% above the baseline of untransfected mMSCs whereas TCF3 did not promote AMY1 expression in mMSCs, as shown in Figure 2A. Neither MIST1 nor TCF3 transfected cells induced the expression of CK19 mRNA. The submandibular gland lysate (mSMX) of 8-week old mice was used as a positive control for qRT-PCR. AMY1 protein expression in MIST1 transfected mMSCs was quantified using WB at 24-h post-transfection (Figure 2B). mMSC overexpressing MIST1 showed an average of a 2.5-fold increase in AMY1 expression, which was normalized by the expression level of GAPDH. A band at 55kDa confirmed the predicted size of AMY1. The band was not found in the cells expressing TCF3, indicating that TCF3 did not induce AMY1. Likewise, neither MIST1 nor TCF3 showed induction of the ductal cell marker CK19 while the positive control, hSGL, clearly showed the expression of CK19.

**Figure 3:**
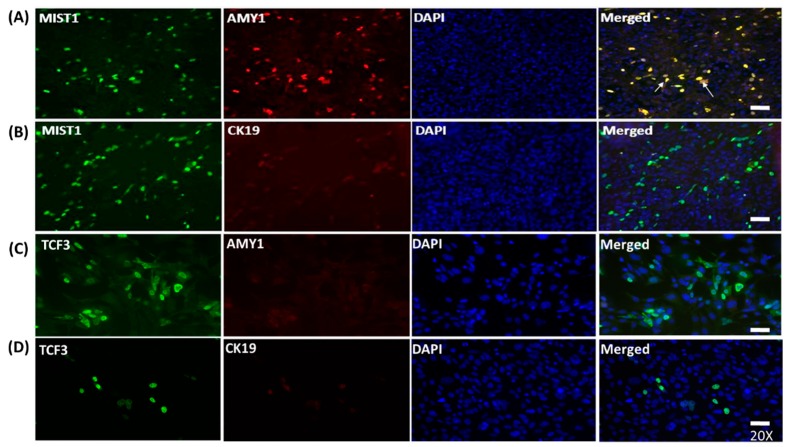
ICC to examine the expression of AMY1 and CK19 salivary gland markers in MIST1 and TCF3 transfected mMSC. MIST1 and TCF3 transfection efficiency was about 28–34% (green). Nuclear localization of MIST1 and TCF3 were confirmed by ICC. (**A**,**C**) MIST1 transfected mMSCs, but not TCF3 transfectants, were also stained positive for AMY1 (red). The merged image demonstrates the co-expression of MIST1 and AMY1 in the same cells (yellow). (**B**,**D**) Neither MIST1 nor TCF3 induced ductal cell marker CK19 expression. Magnification, × 200; scale bar, 20 μm.

The protein expression of AMY1 in MIST1 transfected mMSCs was confirmed by ICC using the transfected cells at 24-h post-transfection. Staining indicated that MIST1 positive mMSCs were also positive for AMY1 (yellow), as indicated with white arrows in the merged image on the top panel of Figure 3A. In contrast, TCF3 overexpression in mMSCs did not induce AMY1 expression (Figure 3C). Furthermore, neither of the two TFs led to CK19 expression (Figure 3C,D). DAPI was used to stain the nucleus.

### 2.3. mMSCs Express AQP5, Which Is Localized to the Nucleus

**Figure 4:**
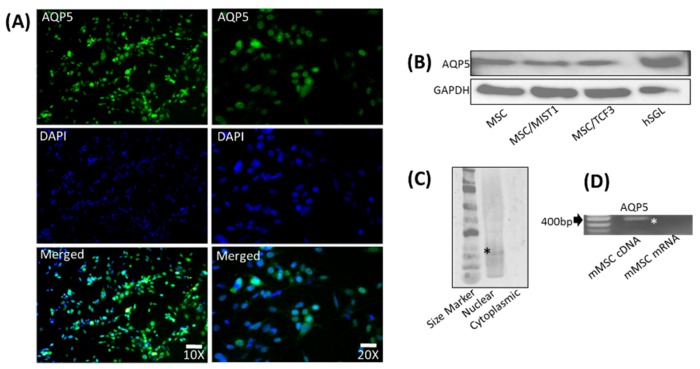
Protein expression of AQP5 in mMSCs localized to the nucleus detected by ICC and WB. (**A**) Ubiquitous expression of AQP5 was detected in mMSCs (green). Images were taken at 100 × and 200 × magnifications. DAPI was used for nuclear staining. Merged images show a co-localization of AQP5 (green) and DAPI in mMSCs. scale bar, 10 and 20 μm. (**B**) WB assay indicated bands representing AQP5 detected equally in MIST1 and TCF3 transfected and untransfected mMSCs, as well as the positive control, hSGL. GAPDH antibody was used for loading control. (**C**) Using Nuclear and cytoplasmic fractionations resolved on a WB, the positive band of AQP5 was detected in the nuclear but not in the cytoplasmic fraction. (**D**) RT-PCR was also performed to confirm mRNA expression of AQP5 in mMSCs. Reverse transcribed cDNA of GAPDH was used as a loading control. (*) indicates the band of interest.

We examined whether the overexpression of MIST1 or TCF3 in mMSCs could lead to the induction of molecules that are critical for saliva secretion. The transfectants were examined for AQP5, which is known to be a water channel protein in the salivary acinar cells, using ICC and were visualized at a 100× and a 200× magnification (Figure 4A). mMSCs demonstrated the positive expression of AQP5. Nuclear localization of AQP5 was observed in the majority of the stained cells in the merged image depicted (Figure 4A). Neither MIST1 nor TCF3 altered AQP5 protein expression in mMSCs, as confirmed by WB (Figure 4B). When we fractionated the transfected cells for WB, only the nuclear fraction showed a positive band for AQP5, while no band was detected in the cytoplasmic fraction (Figure 4C). hSGL was used as a positive control, and GAPDH was used as a loading control. Furthermore, at the gene level, mRNA for AQP5 was detected utilizing RT-PCR with AQP5-specific primers (Figure 4D). AQP5 without reverse transcriptase was negative for AQP5 gene amplification by RT-PCR, confirming no genomic DNA contamination.

### 2.4. mMSCs Express M3R, Which Is Localized to the Endoplasmic Reticulum (ER)

**Figure 5:**
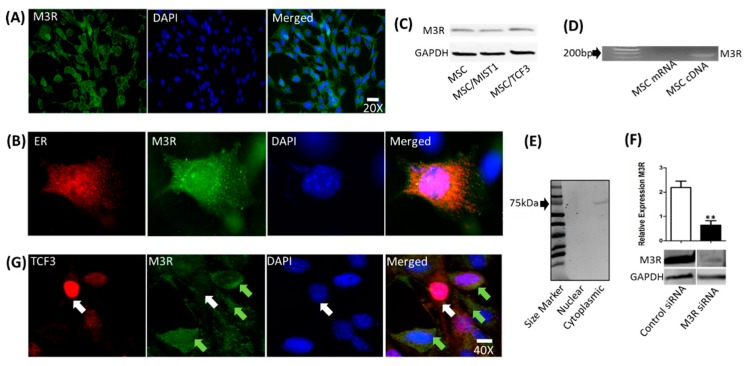
mMSCs express M3R in the cytoplasmic compartment co-localized to the endoplasmic reticulum (ER). (**A**) Generalized expression of M3R (green) localized to the cytoplasmic space of mMSCs, as shown in the top panel. Magnification of × 20; scale bar, 20 μm. (**B**) To confirm M3R localization to the ER, cells were co-stained with antibodies specific to M3R (green) and ER (red). At the single cell level, the merged image confirms the localization of M3R in the ER by the observed orange color (scale bar, 2 μm). DAPI was used for nuclear staining. (**C**) Furthermore, WB was performed to confirm M3R protein expression in transfected and untransfected MSCs. (**D**) RT-PCR was also performed to confirm M3R mRNA expression of MSCs. (**E**) Additionally, nuclear and cytoplasmic protein fractions from mMSCs analyzed by WB confirmed the confinement of M3R to the cytoplasmic compartment. (**F**) Knocking down M3R with a sequence-specific siRNA led to a significant reduction of the M3R level. ** *p* < 0.01 by Student’s *t*-test. (**G**) At 24-h post-transfection, TCF3 expressing mMSCs demonstrated reduced expression of M3R observed in the cytoplasm (white arrows). Cells that have low or no expression of TCF3 maintained M3R expression (green arrows). Magnification of × 40; scale bar, 5 μm.

M3R, which is the major receptor subtype expressed in the salivary gland acinar cells for saliva secretion, was examined in the transfected and non-transfected mMSCs by ICC. At a 20× magnification, we clearly identified the cytoplasmic distribution of M3R protein as demonstrated by the green color (Figure 5A). In the merged image, no overlap between the DAPI and M3R staining was detected, which suggests no nuclear localization for M3R. To confirm the location of M3R, we co-stained M3R and ER and evaluated the fixed cells by ICC. The orange color in the merged image (Figure 5B), suggests that M3R is mainly localized to the ER in the cells. M3R expression observed by ICC was confirmed by WB as shown in Figure 5C. Interestingly, overexpressing MIST1 or TCF3 in mMSCs did not alter the expression of M3R. We also performed RT-PCR to confirm that M3R detected in mMSCs by ICC is indeed the M3R subtype as antibodies can cross-react to other subtypes (see Figure 5D). The result indicates the endogenous expression of M3R in mMSCs. To confirm that purified RNA is not contaminated with genomic DNA, RT-PCR was performed on non-reverse transcribed cDNA, resulting in the absence of M3R expression as expected. To further confirm the location of M3R expression, nuclear and cytoplasmic fractions were blotted using WB. The band at 75 kDa corresponding to M3R detected only in the cytoplasmic fraction confirmed its localization in the cytoplasm (Figure 5E). Knock-down of M3R expression by a sequence-specific siRNA led to an 80% reduction of M3R expression (Figure 5F), confirming its subtype to be M3R. Interestingly, transfectants that strongly overexpressed TCF3 (red) showed lack of M3R expression in the cytoplasm (white arrows) (Figure 5G). However, MSCs with a low expression of TCF3 maintained M3R expression (green arrows).

## 3. Discussion

Although much progress has been made in recent years to identify TFs that drive stem cell differentiation into salivary precursors, no key regulatory TFs have yet been determined. Since our laboratory has identified six TFs by high throughput proteomics in our previous work [2,28], we investigated the roles of MIST1 and TCF3 first in this study in MSC transdifferentiation with AMY1 and CK19 as a marker for acinar and ductal salivary epithelial cells, respectively.

MIST1 is known to be expressed in salivary acinar cells [35]. While MIST1′s function in salivary acinar cell differentiation is not clear, it has been extensively studied in pancreatic exocrine acinar cells. Recent studies have shown that it induces and maintains the pancreatic exocrine acinar cell phenotype such as cell polarity and its knock out leads to apical-basal disorganization, thus resulting in dysfunction of the pancreatic acinar secretory apparatus [35,36]. The most important enzyme produced by the salivary gland is AMY1. It is the first enzyme that begins the process of digestion by breaking complex carbohydrate. Interestingly, it was believed that AMY1 was produced by ductal cells in mouse submandibular glands until the recent study detecting its strong expression in the acinar cells of parotid and weaker expression in submandibular glands of mice [37]. In our present study, we found for the first time that MIST1 alone can upregulate AMY1 expression in mMSCs.

Furthermore, MIST1 is a class B of basic helix-loop-helix (bHLH) TF that forms a homodimer to activate target genes [38,39,40]. In a recent study, MIST1 homodimer promoter binding site has been identified to be the E-box (CATATG) [41]. In analyzing the promoter regions for salivary and pancreatic amylase, only the promoter region of the salivary amylase, AMY1, presented two of this specific binding site. This may explain our finding that MIST1 does not induce pancreatic amylase, AMY2, but AMY1, as shown in our transfected mMSCs. In the pancreas, MIST1 maintains the secretory apparatus while the establishment of the exocrine secretory phenotype requires the pancreatic transcription factor, PTF1α. Both TFs are known to activate over 100 genes in pancreatic exocrine acinar cells [42]. The presence of PTF1α in the mouse submandibular gland (SMG) has not been known until our recent discovery [28]. Thus, it is highly perceivable that co-expressing MIST1 and PTF1α may lead to a robust expression of AMY1 in mMSCs and other markers of salivary precursors, which warrants further investigation. Interestingly, MIST1 has no influence on the expression of the ductal cell marker, CK19, in mMSCs, implying it may play lesser or no rolein ductal cell differentiation or development.

We also tested the effect of TCF3 on mMSC transdifferentiation. TCF3, also known as E2A, plays a role in immunoglobulin gene rearrangement in B-cell development and it is critical in T-cell differentiation and maturation [43,44].However, we didn’t find any effect on salivary gland marker expression in the transfected mMSCs. In another study, E47, one the two TCF3 subunits known for its role in B and T lymphocyte development [45], induced high levels of expression of acinar digestive enzymes and feed-forward activation of the acinar maturation network regulated by MIST1 [46]. However, our testing of TCF3 has no effect on the main digestive enzyme, AMY1, produced by the salivary glands.

In addition to AMY1, M3R is an important marker and signal transduction protein for salivary acinar cell function. During the course of identifying the roles of MIST1 and TCF3 on mMSC transdifferentiation into salivary precursors, we have found that M3R is constitutively expressed in the ER of mMSCs. M3R is a G-protein coupled receptor expressed in salivary acinar cells and responsible for salivary secretion in glandular tissues [47]. It responds to acetylcholine, which leads to a cascade of signal transduction in the cell to secrete saliva [48,49]. To our knowledge, no studies have shown the expression of M3R in mMSCs. Interestingly, another subtype M2R has been reported to be expressed in rat adipose mesenchymal stem cells, responsible for maintaining the cells’ quiescent status [50]. To rule out our subtype detected is M3R, no M2R, we used a M3R sequence-specific siRNA and knocked down its expression in mMSCs, confirming the subtype of M3R. Since M3R expression in our mMSCs was found to be located in the ER mainly, it may not contribute to signal transduction for secretion during the quiescent status of mMSCs. In fact, when we stimulated mMSCs with an MR agonist, carbachol, the relocation of AQP5 was not observed (data not presented), indicating M3R expression on the cell membrane is important for AQP5 translocation. AQP is known to be translocated to the membrane for the secretion of saliva upon stimulation of M3R [4]. Therefore, it is presumed that at the time of functional differentiation, the membrane localization of M3R would be critical for salivary secretion in response to M3R agonists or acetylcholine.

Our current study indicates that overexpression of MIST1 did not alter M3R expression in mMSCs. This is consistent with a recent study that reported a minimal effect of MIST1 knockout on M3R expression in mouse pancreatic acinar cells [34]. However, we found that TCF3 may play an inhibitory effect on M3R since its overexpression downregulates M3R in mMSCs as shown in ICC. TCF3 is known to play an inhibitory role in embryonic stem cell differentiation [37]. Consistently, the fact that TCF3 had no direct effect on AMY1 expression may underscore its general inhibitory role in differentiation. Therefore, initial upregulation of TCF3 in differentiating mMSCs during co-culture may reflect its role in promoting stemness while suppressing differentiation.

In our study, AQP5 was detected in mMSCs. However, neither MIST1 nor TCF3 has influenced its expression level or location. AQP5 has not been well studied in the bone marrow stem cells in terms of expression and function. Two studies have shown the enhanced expression of AQP5 in bone marrow aspirates during hypoxic lung injury [51,52]. Interestingly, we detected AQP5 to be expressed in mMSCs nuclei. A study indicates that its localization of AQP5 to the plasma membrane leads to a reduction in the differentiation capacity in mMSCs [53]. The availability of key TFs would be important for proper localization of AQP5 for salivary acinar cell functions. It’s trafficking to the plasma membrane requires activation of the M3R receptor on the cell membrane as mentioned earlier. A ligand binding to M3R leads to intracellular Ca^2+^ release from ER and thus, resulting in trafficking of AQP5 to the apical plasma membrane [4,54,55]. In addition to the plasma membrane trafficking of AQP5, it may also be directed to the nucleus to maintain cellular homeostasis in rat parotid acinar cells [56]. So, its location in the nucleus of mMSCs is important for the cell internal homeostasis and would require M3R in the membrane for activation in the differentiated salivary precursor cells.

In our present study, we have shown that mMSCs express two of the most important proteins for salivary gland function and signal transduction, M3R and AQP5. We identified that mMSCs constitutively express M3R and that they have the capacity to enhance AMY1 expression with a single TF such as MIST1. Furthermore, TCF3 provides negative regulation for M3R expression when it is overexpressed in mMSCs in contrast to its function in T and B cells, but consistently with its function in embryonic stem cells. Our findings substantiate mMSCs as a potentially attractive stem cell model for their plasticity into salivary precursors. Further studies are warranted to identify key co-regulators to promote cellular and functional differentiation, especially in a poor microenvironment of chronic inflammation or radiation damage. Functional assays of transfected cells with additional TFs such as PTF1α may provide better insights into master regulatory networks needed for the reprogramming of mMSCs.

## 4. Limitations and Future Directions

Data from our work have indicated the potential role of MIST1in inducing salivary acinar cell marker, AMY1, gene expression in mMSC. As multiple genes have been reported to be regulated by MIST1 in pancreatic acinar cells [37], we anticipate that MIST1 influences numerous genes in mMSC during the process of MET. Further studies will identify the downstream target genes of MIST1 via high-throughput approachesA mechanism by which MIST1 induces AMY1 will also be investigated. 

Future in vivo studies will add valuable information as to determine if direct MET of mMSCs by MIST1 can generate functional acinar cells that are capable of secreting amylase and/or fluid. Generally, a secretory apparatus in secretory glands requires environmental cues from the surrounding matrix for it to be functional. Thus, delineating complex interactions among endogenous TFs and exogenous signals that lead to functional differentiation of mMSCs will be of the utmost importance in our future studies. 

## 5. Materials and Methods

### 5.1. Mesenchymal Stem Cell Culture

mMSCs were purchased from Life Technologies, Inc. They were harvested from the bone marrow of C57BL/6 mice at less than 8 weeks of age. The manufacturer reported purity of >95% of a positive expression of stem cell markers, CD29+, CD44+, CD34+, Sca1+, and a demonstrated ability to differentiate into multiple cell types such as adipocytes, chondrocytes, and osteocytes in vitro. We verified their phenotype by western blotting (WB) using antibodies for Thy1, Sca1, and CK45 (data not shown). mMSCs were thawed and cultured in T-75 tissue culture flasks containing 15 mL of DMEM/F12 with 10% stem cell-qualified FBS and 1 μg/mL of penicillin/streptomycin. Cells were incubated in 5% CO_2_ at 37 °C. mMSCs were passaged using 0.05% trypsin-EDTA (Thermo Fisher Scientific Waltham, MA, USA) at 80–90% confluence. All experiments used mMSCs with passages between 3 to 6 after thawing.

### 5.2. Recombinant MIST1 and TCF3 Expression

Full-length mouse MIST1 and TCF3 clones were purchased from (Dharmacon, Lafayette, CO, USA) in pCMV-SPORT6 plasmid with clone IDs of 4165423 and 2631291, respectively. The fidelity of these cDNAs was confirmed by gene sequencing. Inserted ORF sequence of each TF was amplified using the designated restriction sites by PCR and inserted into the multiple cloning sites of HindIII and XhoI in pcDNA3.1/cMyc/BioID plasmid (Addgene plasmid # 35700). Phusion master mix (Thermo Fisher Scientific, Waltham, MA, USA) was used for the PCR amplification and T4 ligase (Promega, Madison, WI, USA) was used for the construction following the manufacturers’ instruction.

### 5.3. Transient Transfection

For a 6-well plate for WB, we used mMSCs at a confluence of 70–80%. We incubated 5.7 μg of DNA from the respective constructs with 5.9 μL Lipofectamine Stem Transfection Reagent (Thermo Fisher Scientific, Waltham, MA, USA) to a volume of 200 μL of Opti-MEM culture. This was incubated for 15 min at room temperature. The mix was then added to the cells incubated in 2 mL of DMEM/F12 with 10% MSC-qualified FBS. Cells were then collected for WB at 24 h post-transfection. For 8-chamber slides for immunocytochemistry (ICC), approximately 2 × 10^4^ cells were seeded in each chamber of the 8-well chamber slides (Thermo Fisher Scientific, Waltham, MA, USA). We mixed 2.3 μg of DNA from the respective construct with Lipofectamine Stem Transfection Reagent (Thermo Fisher Scientific, Waltham, MA, USA) to a volume of 50 μL of Opti-MEM culture for 15 min at room temperature. The mixture was added to the cells and gently mixed with 300 μL of DMEM/F12 with 10% MSC-qualified FBS.

### 5.4. RNA Isolation, RT-PCR, and Quantitative Real-Time PCR (qRT-PCR)

Total RNA was extracted from cells by using Trizol reagent (Thermo Fisher Scientific, Waltham, MA, USA). Reverse transcription (RT) of 2 µg total RNA was performed with the cDNA Reverse Transcription Kit (Promega, Madison, WI, USA) according to the manufacturer’s protocol. We used SYBR Green master mix (Thermo Fisher Scientific Waltham, MA, USA) containing dNTPs and thermostable hot-start DNA polymerase. We calculated relative expression to the internal control, 18S rRNA, using 2^-∆∆Ct^ method [57]. For forward primers we used: 18S rRNA: cggctaccacatccaaggaa, MIST1: tggtg gctaaagcta cgtgt, TCF3: agtgagcgga atgcctatgc c, AMY1: gattccaact gcagtggaag gc, AQP5: tgctggagca ggcatcctgt ac, CK19: tgacaatgct cgcctggctg c, M3R: tagagcggact ggacagtccg. Reverse primers were: 18S rRNA: gctggaattaccgcggct, MIST1: tggt cttcatagct ccaggctgg, TCF3: gtatctgcca ggccaccatc tg, AMY1: agctaagta tctctcacat tcc, AQP5: agatta actccaccac cacgg, CK19: cttcaggctc tcaatctgca tc, M3R: actcaatcca cagtccactg ag.

For RT-PCR, 200 ng of isolated RNA were subjected to Phusion Flash High-Fidelity PCR Master Mix (Thermo Fisher Scientific Waltham, MA, USA) with the presence of isotype-specific designed primers for M3R and AQP5 amplification. The reaction consisted of a temperature of 98 °C for 2 min, 35 cycles of 98 °C for 8 s, 52 °C for 30 s, 72 °C for 30 s. Products were run on 1% agarose gel for 30 min. Forward primers: AQP5, cgtggtggtggagttaatctt; M3R, ctcagtggactgtggattgagt, and reverse primers: AQP5, gtcagctcgatggtcttcttc; M3R, gaggttgtactgttactgtgc.

### 5.5. Western Blot (WB) Analysis

At 24-h post-transfection, a commercially available detergent-based cell extraction buffer RIPPA (Thermo Fisher Scientific Waltham, MA, USA) supplemented with protease inhibitor cocktail (Roche) was used to lyse cells. The Bicinchoninic Acid assay (BCA assay) was utilized to measure protein concentration. Samples were loaded in Laemmli buffer and electrophoresed under reducing conditions on 4–20% Tris-Glycine TGX gels (Bio-Rad, Hercules, CA, USA). Proteins were transferred to PVDF membranes (Bio-Rad) utilizing semi-dry transfer apparatus (Thermo Fisher Scientific, Waltham, MA, USA), and blots were blocked with 5% non-fat dry milk in TBS-Tween for one hour. Rabbit polyclonal anti-MIST1 antibody (Abcam Cambridge, MA, USA, ab107390), rabbit polyclonal anti-TCF3 antibody (Santa Cruz Biotechnology, Dallas, TX, USA, sc763), goat polyclonal anti-cMyc antibody (Bethyl, A190-104A,), chicken polyclonal anti-BirA antibody (a gift from Dr. YJ Park), rabbit polyclonal anti-AMY1 antibody (Biomatik CAU25843), rabbit polyclonal anti-M3R antibody (Abcam, Cambridge, MA, USA, AB126168), rabbit polyclonal anti-AQP5 (Abcam, Cambridge, MA, USA, Ab78486), rabbit polyclonal anti-CK19 (Abcam, Cambridge, MA, USA, ab52625), and goat anti-parotid secretory protein (PSP) antibody (Everest EBP10621) all with a 1:500 dilution rate, were added to the blocking solution and incubated with blots overnight at 4 °C. An anti-rabbit, goat, or chicken HRP-conjugated antibody (1:5000) (cell signaling) was used as a secondary antibody for 2 h at room temperature followed by detection with Chemiluminescent Substrate (Promega Corporation, Madison, WI, USA). Membranes were visualized with the ECL detection system (Fisher). GAPDH antibody (Thermo Fisher Scientific, Waltham, MA, USA) was used as a loading control, and secondary antibody and chemiluminescent detection were completed as above. We used human salivary gland lysate (hSGL) (Santa Cruz Biotechnology, Dallas, TX, USA) as a positive control.

### 5.6. Immunocytochemistry (ICC)

Cells were first fixed with 4% paraformaldehyde for 20 min at room temperature. Following the removal of the fixative by washing with PBS, cells were treated with cold methanol for 20 min at −20 °C for permeabilization. Five percent FBS (Thermo Fisher Scientific, Waltham, MA, USA) in PBS was applied for 2 h to block unspecific binding. Primary antibodies for MIST1 (Abcam, ab107390,1:100), rabbit polyclonal anti-TCF3 antibody (Santa Cruz, sc763, 1:100), goat polyclonal anti-cMyc antibody (Bethyl, A190-104A, 1:100), rabbit polyclonal anti-AMY1 antibody (Biomatik, CAU25843, 1:100), anti-rabbit polyclonal anti-M3R antibody (Abcam, ab126168, 1:100), rabbit polyclonal anti-AQP5 (Abcam, Ab78486, 1:100), and anti-rabbit polyclonal anti-CK19 antibody (Abcam, ab52625, 1:100) were diluted in 5% FBS blocking buffer and applied on cells overnight at 4 °C. An anti-rabbit/goat Alexa Fluor™ 568 or 488 nm conjugated antibodies (1:200) was used as a secondary antibody for 2 h at room temperature followed by a final wash. Endoplasmic reticulum staining was performed by an ER-GFP, BacMam 2.0 labeling kit (Cell light, C10590). Images were obtained using a Zeiss Axiovert 200M microscope equipped with a AxioCam MRm camera and AxioVs40 software (Ver. 4.7.1.0) (Zeiss, Oberkochen, Germany).

### 5.7. M3R Protein Knockdown by siRNA

M3R siRNAs were purchased from Dharmacon, Inc. (D-042172-01-0002) along with the irrelevant siRNA (D-001210-01-05) (Dharmacon, Lafayette, CO, USA). We plated 2 × 10^5^ mMSCs per well in a 6-well plate. At 24-h post-transfection, we used DharmaFECT-1 transfection reagent (Dharmacon, Lafayette, CO, USA) to transfect mMSCs with 25 nM of siRNAs in 200 µL Opti-MEM (Thermo Fisher Scientific, Waltham, MA, USA), following the manufacturer’s recommendations. The mixture was incubated at room temperature for 15 min. Each of the combined mixtures was then added to 1.6 mL of antibiotic-free growth medium in 5 mL tubes (Falcon), mixed well, and added to the cells. After 24-h incubation, the medium was replaced with complete medium containing antibiotics. Cells were then grown for an additional 24 h. Whole protein lysates (50 µg) were used for WB. The primary antibodies were an anti-M3R antibody (Abcam, Cambridge, MA, USA) at 1:500 and anti-GAPDH antibody (Thermo Fisher Scientific, Waltham, MA, USA) at 1:1000. Secondary antibody conjugated to IRDye800CW (Li-Cor Biosciences, Lincoln, NE, USA) was applied at 1:1000 for detection. Images were acquired with the Odyssey Infrared Imaging System (Li-Cor Biosciences, Lincoln, NE, USA). Protein expression was quantified with the ImageJ program (NIH).

### 5.8. Statistical Analysis

Each experiment was conducted in duplicate and run independently at least 3 times. A student’s *t*-test using Prism 5 (GraphPad Software, Inc., San Diego, CA, USA) was performed where appropriate. Asterisks *, **, and *** indicate statistical significance levels of *p* < 0.05, *p* < 0.01, and *p* < 0.001, respectively. Error bars represent the SEM.

## Abbreviations

MSCs	Mesenchymal stem cells
SjS	Sjögren’s syndrome
mMSCs	mouse bone marrow-derived MSCs
TFs	transcription factors
AMY1	alpha-salivary amylase 1
CK19	cytokeratin19
MET,	mesenchymal-epithelial transition
MIST1	muscle, intestine and stomach expression-1
TCF3	transcription factor E2a
PTF1α	pancreas-specific transcription factor 1 α
ASCL3	achaete-scute complex homolog 3
ANKRD56	ankyrin repeat domain-containing protein 56
HMG20B	high mobility group protein 20B
M3R	muscarinic type 3 receptor
AQP5	aquaporin 5
WB	western blot
ICC	Immunocytochemistry
RT-PCR	reverse transcription PCR
qRT-PCR	quantitative real-time PCR
